# Sustainable KCl‐Assisted PbI_2_ Recycling for High‐Performance Quantum Dot Solar Cells

**DOI:** 10.1002/advs.202522107

**Published:** 2026-02-21

**Authors:** Jihong Lan, Qiang Zeng, Xinwei Guan, Linhong Li, Songyan Yin, Sandhuli S Hettiarachchi Dehigaspitiya, Guozheng Shi, Kou Li, Shujuan Huang, Fangyang Liu, Dewei Chu, Long Hu

**Affiliations:** ^1^ School of Chemistry and Materials Engineering Xinxiang University Xinxiang Henan China; ^2^ School of Metallurgy and Environment Central South University Changsha China; ^3^ School of Engineering Macquarie University Sydney New South Wales Australia; ^4^ Solid State and Elemental Analysis Unit Mark Wainwright Analytical Centre Surface Analysis Laboratory University of New South Wales (UNSW) Sydney New South Wales Australia; ^5^ School of Materials Science and Engineering University of New South Wales (UNSW) Sydney New South Wales Australia

**Keywords:** colloidal quantum dots, green manufacturing, lead recycling, lead sulfide, metallurgy, solar cells

## Abstract

Lead‐based semiconductors play a critical role in emerging high‐performance optoelectronics. For scalable, cost‐effective fabrication, recycling lead waste from spent lead‐acid batteries has proven a promising strategy. Nevertheless, achieving device‐grade semiconductor quality from recycled sources remains a critical unresolved challenge. Here, we present the first additive‐assisted lead recycling strategy, in which trace amounts of KCl are introduced during the recycling process to enable the formation of high‐purity PbI_2_. The incorporated KCl enhances crystallization and removes impurities, while residual KCl further improves the colloidal stability and photophysical properties of PbS quantum dots (QDs), addressing key challenges in their direct synthesis. The resulting PbS QD solar cells with KCl additive achieved a power conversion efficiency of 13.3%, surpassing those fabricated with commercial PbI_2_ (12.1%) and recycled PbI_2_ without the additive (11.0%). Moreover, CsPbI_3_ QDs synthesized from KCl‐assisted PbI_2_ exhibited enhanced surface integrity, stronger photoluminescence, and longer carrier lifetimes, and the resulting solar cells delivered a superior efficiency of 16.6%, outperforming devices fabricated from commercial PbI_2_ (15.4%), demonstrating the universality of this approach across different lead‐based QD systems. This work establishes a sustainable, additive‐assisted metallurgy‐semiconductor coupling strategy, providing an energy‐efficient pathway to convert lead waste into high‐purity semiconductor precursors for advanced optoelectronic devices.

## Graphical abstract

An additive‐assisted lead recycling strategy is developed by introducing trace KCl during PbI_2_ recrystallization from spent lead‐acid batteries. KCl regulates crystallization and impurity removal, yielding high‐purity PbI_2_ and enabling the formation of colloidally stable PbS and CsPbI_3_ quantum dots. As a result, PbS and CsPbI_3_ quantum dot solar cells achieve enhanced efficiencies of 13.3% and 16.6%, respectively.

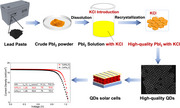

## 1 Introduction

Lead (Pb) sources play a crucial role in the development of emerging high‐performance optoelectronic materials, including lead halide perovskites and infrared lead chalcogenide quantum dots (QDs) [1, 2, 3, 4, 5, 6, 7, 8]. However, the preparation of Pb precursors through conventional metallurgical routes, such as pyrometallurgical smelting [9], vacuum crystallization [10], and chemical vapor deposition [11], remains energy‐intensive and carbon‐heavy, requiring high temperatures and producing significant carbon emissions and waste. As a sustainable alternative, recycling Pb from spent lead‐acid batteries has recently attracted increasing attention as an economically viable and environmentally responsible approach to reduce both material cost and industrial pollution [12, 13, 14, 15, 16].

Recycled Pb sources have been successfully employed in various optoelectronic devices, such as solar cells [17, 18, 19] and photodetectors [20, 21]. Nevertheless, their device performance generally lags behind that of counterparts fabricated from commercial Pb precursors, primarily due to impurity incorporation, stoichiometric inhomogeneity, and limited crystallinity. These intrinsic deficiencies severely hinder the reproducibility and scalability of recycled Pb‐based semiconductors, particularly for high‐end or precision optoelectronics requiring high material purity [22, 23, 24]. In this context, developing a facile and scalable recycling strategy capable of delivering device‐grade Pb precursors with quality comparable to or exceeding that of commercial sources remains an urgent and unresolved challenge.

To enhance the optoelectronic quality and stability of Pb‐based semiconductors, elemental doping has emerged as an effective and versatile strategy [25, 26, 27, 28, 29, 30, 31, 32, 33]. For instance, Cd incorporation into Pb halide perovskites was reported by Sargent's group to suppress defect density, boosting power conversion efficiency (PCE) and device stability [27]. Our previous work revealed that Ag doping can modulate carrier concentration and transport dynamics in mixed‐halide perovskites, leading to improved charge extraction, PCE, and photostability in Pb‐based solar cells [28, 29]. Similarly, Rb incorporation in inorganic Pb‐based perovskites refines crystal symmetry by partially substituting Cs, thereby stabilizing the lattice and optimizing electronic coupling [30, 31]. Among various dopants, potassium (K) has recently shown synergistic multifunctionality, capable of concurrently suppressing defect density, passivating surface traps, and stabilizing crystal phases in a wide range of Pb‐based semiconductors, including lead sulfide (PbS) colloidal quantum dots (CQDs) [34, 35], perovskite thin films [36], and perovskite nanocrystals [37, 38]. Inspired by these advances, we hypothesize that deliberate K‐based additive incorporation during Pb recycling could simultaneously promote precursor purification and endow the resulting semiconductors with improved electronic and structural integrity.

In this work, we report a low‐temperature, solution‐processed method that efficiently converts lead paste from spent batteries into high‐purity PbI_2_ by introducing a potassium (K)‐based additive. During recrystallization, trace amounts of potassium chloride (KCl) were added to the PbI_2_ in *N,N*‐dimethylformamide (DMF) solution, promoting crystal growth and impurity removal during antisolvent precipitation with isopropanol. Residual KCl in the recycled precursor further modulated the chemical environment and passivated surface defects in the resulting PbS CQDs, leading to markedly improved colloidal stability and carrier dynamics. This advancement directly addresses a key challenge in the emerging direct synthesis of PbS CQDs, enabling device‐grade material quality from recycled sources. The resulting PbS CQD solar cells achieved a PCE of 13.3%, surpassing those fabricated with commercial PbI_2_ and with recycled PbI_2_ without the additive. Furthermore, the same KCl‐assisted PbI_2_ precursor was successfully used to synthesize CsPbI_3_ CQDs, yielding narrower size distributions, improved surface integrity, and longer carrier lifetimes than those of commercial counterparts. Therefore, the resulting CsPbI_3_ CQD solar cells with KCl achieved a champion efficiency of 16.6%, outperforming the 15.4% champion efficiency of commercial CsPbI_3_ QD devices, confirming the universal benefit of KCl incorporation. Overall, our additive‐assisted recrystallization strategy bridges green metallurgy and semiconductor engineering, offering a versatile, energy‐efficient, and sustainable route for recycling Pb waste into high‐purity precursors for high‐performance optoelectronic materials. Beyond advancing wet‐processing metallurgy, this work establishes a general approach for transforming industrial waste into functional semiconductors, contributing to next‐generation sustainable electronics.

## 2 Results and Discussion

### 2.1 Additive‐Induced Pb Source Recycling from Spent Batteries

**FIGURE 1:**
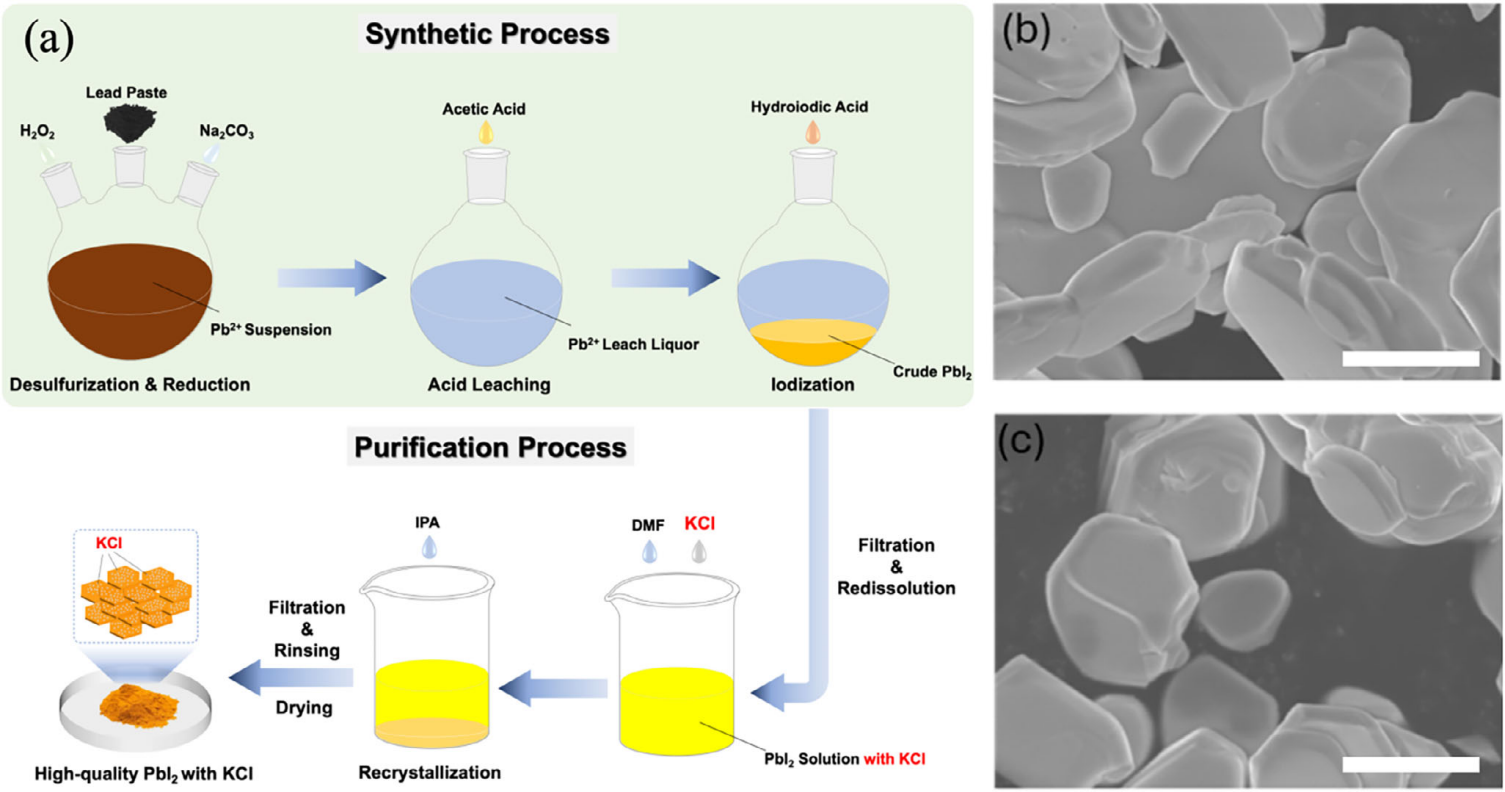
(a) Schematic illustration of the KCl‐assisted PbI_2_ recycling process (PbI_2_‐K). SEM images of (b) PbI_2_‐K and (c) PbI_2_‐W. Scale bar: 1 µm.

The recycling process used in this work was modified from that reported in our previous work [14, 22]. Figure 1a schematically illustrates the overall procedure for Pb‐source recovery from spent lead–acid batteries assisted by a KCl additive. The lead paste, composed primarily of PbSO_4_, PbO_2_, and PbO, was first collected by dismantling spent batteries, serving as the raw Pb source for recycling. The paste was then dispersed in deionized water and subjected to sequential desulfurization and reduction reactions. Specifically, aqueous Na_2_CO_3_ solution and 30 wt% H_2_O_2_ were subsequently added to convert PbSO_4_ to PbCO_3_ and PbO_2_ to PbO, respectively. Afterward, excess acetic acid was added to the suspension to leach Pb^2+^ ions, yielding a clear lead acetate solution. This solution was then heated to 90°C to remove residual H_2_O_2_. After cooling to room temperature, hydroiodic acid was added dropwise while stirring, resulting in the gradual precipitation of yellow PbI_2_. The crude PbI_2_ powder was collected by filtration, thoroughly rinsed, and dried.

For further purification, the crude solid was redissolved in DMF to form a saturated solution, into which a trace amount of KCl additive (with a KCl: PbI_2_ molar ratio of 1:100) was introduced to promote PbI_2_ crystallization. Since KCl has low solubility in DMF (≈0.1 g/100 mL) [39] and extremely low solubility in IPA (≈0.0023 g/100 mL) [40], KCl would be precipitated first during the recrystallization. More importantly, KCl can interact with PbI_2_ at the molecular level, leading to interactions with the PbI_2_ lattice. These interactions prevent “flash” crystallization, thereby generating fewer crystal seeds. Due to reduced PbI_2_ seeds, this would benefit the slow growth of PbI_2_ crystals and the expulsion of impurities. Therefore, high‐quality PbI_2_ crystals (denoted PbI_2_‐K) could be obtained through precipitation by the addition of sufficient isopropanol (IPA), followed by filtration, rinsing, and vacuum drying at 60°C overnight. For comparison, a control sample of PbI_2_ was prepared using the same procedure but without the KCl additive, referred to as PbI_2_‐W.

PbI_2_‐K and PbI_2_‐W powders were examined using scanning electron microscopy (SEM). As shown in Figure 1b,c, the PbI_2_‐K sample exhibited significantly larger grain sizes compared to PbI_2_‐W, indicating that the KCl additive effectively promoted crystallization during IPA antisolvent precipitation. X‐ray diffraction (XRD) analysis further confirmed enhanced crystallinity in PbI_2_‐K, as evidenced by stronger, sharper diffraction peaks and smaller full width at half maximum (FWHM) relative to PbI_2_‐W (see Figure S1a,b). These results collectively demonstrate that KCl incorporation facilitates the growth of highly crystalline PbI_2_. Moreover, X‐ray photoelectron spectroscopy (XPS) was performed to verify the elemental composition of both samples. Figure S1c shows the survey spectra, while Figure S1d,e displays the core XPS spectra of K 2p and Cl 2p, which account for 0.83% and 1.04% of atomic ratios in PbI_2_ product with additive, respectively. These values are consistent with the nominal mixing ratio of 1:100 of KCl:PbI_2_ during recrystallization. Inductively coupled plasma mass spectrometry (ICP‐MS) was further employed to quantify elemental contents in PbI_2_‐K, PbI_2_‐W, and commercial PbI_2_ (PbI_2_‐C, Sigma‐Aldrich, 99.99%). As summarized in Table S1, most impurity elements in PbI_2_‐K, such as Ca and Sb, were substantially reduced, while K and Cl contents remained intentionally higher than in PbI_2_‐W and PbI_2_‐C, confirming the successful incorporation of the additive. The calculated purity of PbS‐K is 99.99%, which is comparable to that of the commercial product.

Finally, cost analysis demonstrated that the recycled PbI_2_ product could be obtained at approximately 1.65 USD per gram, an order of magnitude lower than the ≈10 USD per gram price of commercial PbI_2_ from Sigma‐Aldrich company (99.99% purity). This remarkable reduction underscores the economic and environmental advantages of the KCl‐assisted recycling strategy and establishes a scalable route for sustainable Pb‐based semiconductor manufacturing. The details of the cost calculation are provided in the Supporting Information.

### 2.2 PbS CQDs Synthesized Using Recycled PbI_2_

PbS CQDs possess unique advantages for optoelectronic applications, including size‐tunable absorption extending into the infrared (IR) spectrum, multiple exciton generation, and solution processability. Recently, Ma and coworkers initially developed a one‐step, room‐temperature direct synthesis of PbS CQD conductive inks using PbI_2_ precursors [41]. These directly synthesized CQDs are epitaxially capped with PbI_2_, allowing good dispersion in polar solvents such as DMF and establishing a facile, low‐cost pathway for PbS CQD preparation with substantial savings in both time and materials [42]. Unfortunately, PbI_2_‐capped CQDs are prone to regrowth, leading to oversized, polydisperse nanoparticles with broadened energy distributions and severe photocarrier trapping and recombination [42, 43]. To improve colloidal stability, post‐synthetic surface engineering using NH_4_I treatment has been developed to replace weakly bound PbI_2_ ligands, yielding improved surface passivation [42]. Based on this approach, PbS CQD solar cell minimodules with an active area of 12.6 cm^2^ have achieved certified efficiencies of ≈10%. Nevertheless, the additional surface treatment increases complexity and compromises the inherent simplicity and flexibility of direct synthesis. Additionally, commercial lead sources, such as PbI_2_ for PbS CQD synthesis, are typically produced via high‐temperature fire‐smelting, in which metallic Pb reacts with iodine vapor at 500–700°C. This process is highly energy‐intensive and results in a production cost of approximately 10 USD g^−1^ (TCI) [44]. Such a high precursor cost significantly limits the scalability and commercialization potential of PbS CQD technologies.

Here, we develop an in situ stabilization strategy that eliminates the need for post‐synthetic treatments by using KCl‐assisted PbI_2_ as a precursor to produce low‐cost PbS CQDs for high‐performance solar cells. PbS CQDs were synthesized following a previously reported procedure [42]. Specifically, 2.3 g of recycled PbI_2_ with KCl additive, 0.23 g of *N*,*N*′‐diphenylthiourea (DPhTA), and 8 mL of DMF were loaded into a three‐neck flask under inert conditions. After complete dissolution under continuous stirring, the solution was cooled in an ice bath, and 1 mL of butylamine (BTA) was swiftly injected. After 10 min of reaction, a dark colloidal dispersion of PbS CQDs was obtained. For comparison, PbS CQDs were synthesized using PbI_2_‐W and commercial PbI_2_‐C precursors under identical conditions. The resulting samples were denoted PbS‐K, PbS‐W, and PbS‐C, corresponding to the use of PbI_2_‐K, PbI_2_‐W, and PbI_2_‐C, respectively. All CQDs were purified and finally dispersed in DMF for structural characterization and device fabrication.

**FIGURE 2:**
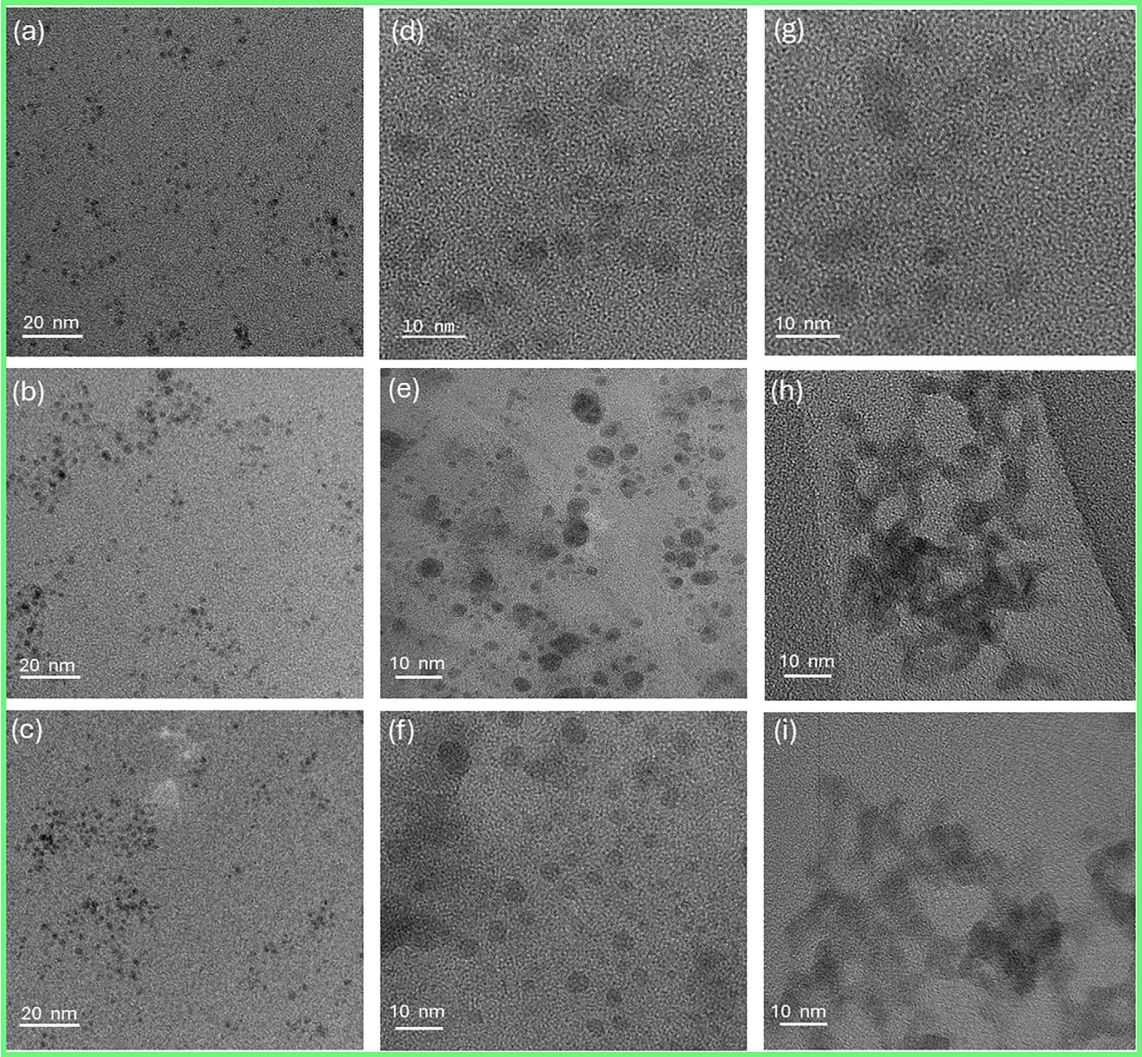
TEM images of PbS CQDs at different storage times. Freshly prepared samples: (a) PbS‐K, (b) PbS‐W, and PbS‐C CQDs. After 48 hours of aging: (d) PbS‐K, (e) PbS‐W, and PbS‐C. After 72 hours of aging: (g) PbS‐K, (h) PbS‐W, and (i) PbS‐C.

Transmission electron microscopy (TEM) was employed to investigate the size distribution and temporal evolution of PbS CQDs to evaluate their colloidal stability. Figure 2a–c shows TEM images of PbS‐K, PbS‐W, and PbS‐C CQDs, exhibiting average QD sizes of 3.6 ± 0.2, 3.7 ± 0.3, and 3.8 ± 0.3 nm, respectively, which are consistent with reported values for this synthesis route. All samples initially displayed uniform size distributions and good dispersion. However, as shown in Figure 2d–f, notable morphological differences emerged after 48 hours of storage: The PbS‐K sample retained its morphology and size, while PbS‐W and PbS‐C gradually evolved into larger, polydisperse nanoparticles. After 72 hours (Figure 2g–i), PbS‐K maintained its original morphology, whereas PbS‐W and PbS‐C underwent severe aggregation, forming interconnected microstructures, consistent with previous observations [42]. We therefore propose that potassium cations act as mobile counter‐ions that electrostatically compensate negatively charged iodide‐based surface ligands. Rather than forming direct chemical bonds or entering the PbS lattice, K^+^ ions are proposed to reside in the diffuse layer near the QD surface, forming an electrical double‐layer‐like structure. Chloride anions play a complementary role by partially coordinating to undercoordinated surface Pb^2+^ sites and participating in mixed‐halide (I^−^/Cl^−^) surface passivation. Compared with iodide, Cl^−^ binds more strongly to Pb^2+^, improving surface stoichiometry and reducing labile Pb‐rich sites that can promote aggregation. In addition, Cl^−^ moderates PbI_2_ solubility in DMF, suppressing ligand desorption and dynamic surface reconstruction. This dynamic charge screening suppresses interparticle Coulombic attraction and improves colloidal stability. While the above considerations explain the K^+^/Cl^−^‐assisted colloidal stabilization, the precise microscopic interactions at the QD‐ligand‐solvent interface remain to be fully elucidated. Further systematic investigations are required to clarify the detailed coupling between electrostatic stabilization, halide coordination, and growth dynamics. These results clearly demonstrate that KCl incorporation during precursor recycling markedly improves the colloidal stability of PbS CQDs, effectively suppressing regrowth and aggregation compared with those synthesized from PbI_2_‐W and PbI_2_‐C.

To further verify the in situ stabilization induced by KCl in precursors, the same KCl‐to‐precursor ratio (1:100) was introduced into the PbS‐C QD DMF solution, and TEM measurements were conducted to monitor the morphological evolution over time. As shown in Figure S2a,b, the sample of PbS‐W with KCl post‐synthetic treatment after 48‐hour storage evolved into large‐sized particles, which is consistent with the PbS‐W and PbS‐C samples. The above results indicate that KCl post‐synthetic treatments cannot efficiently stabilize colloidal PbS QDs.

**FIGURE 3:**
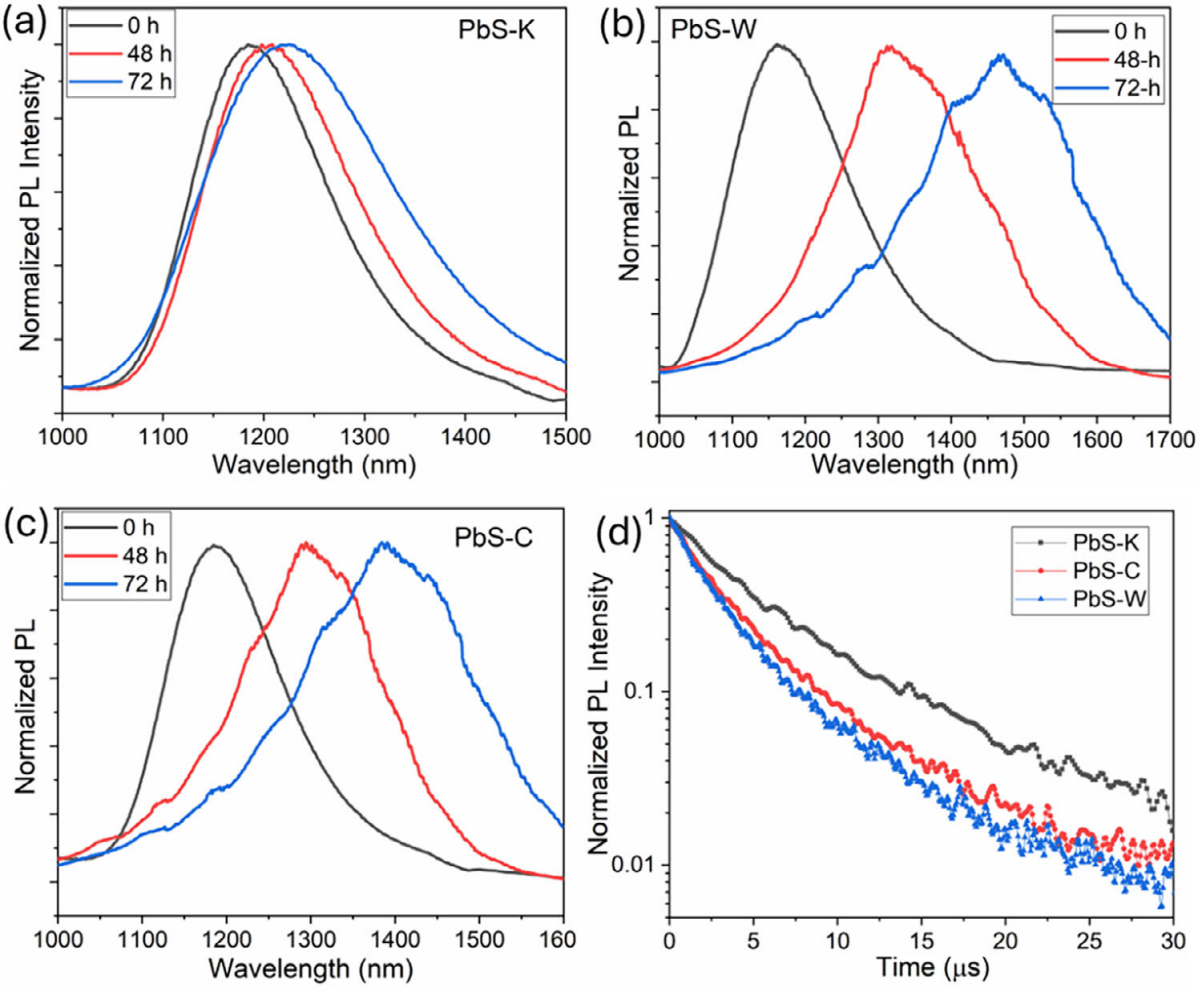
Steady‐state PL spectra of (a) PbS‐K, (b) PbS‐C, and (c) PbS‐W CQDs measured over storage time. (d) TRPL decay curves of the three types of PbS CQDs.

Steady‐state photoluminescence (PL) measurements were performed to further assess the colloidal and optical stability of the PbS CQDs. The PL spectrum of PbS‐K (Figure 3a) exhibited only a slight red shift and minimal broadening, indicating that the particle size and morphology remained largely unchanged, consistent with the TEM observations. In contrast, PbS‐W and PbS‐C (Figure 3b,c) displayed pronounced spectral variations, evolving from a single narrow peak to multiple broadened features, suggesting the formation of polydisperse size distributions in both samples, in agreement with previous reports [45, 46]. Time‐resolved PL (TRPL) measurements were also carried out to investigate carrier dynamics in freshly prepared CQDs (Figure 3d). The average carrier lifetimes were estimated to be 4.1 µs for PbS‐K, 1.8 µs for PbS‐W, and 2.9 µs for PbS‐C, respectively. The notably prolonged lifetime of PbS‐K confirms that KCl incorporation effectively suppresses defect‐mediated nonradiative recombination, hence enhancing carrier relaxation and optical stability.

**FIGURE 4:**
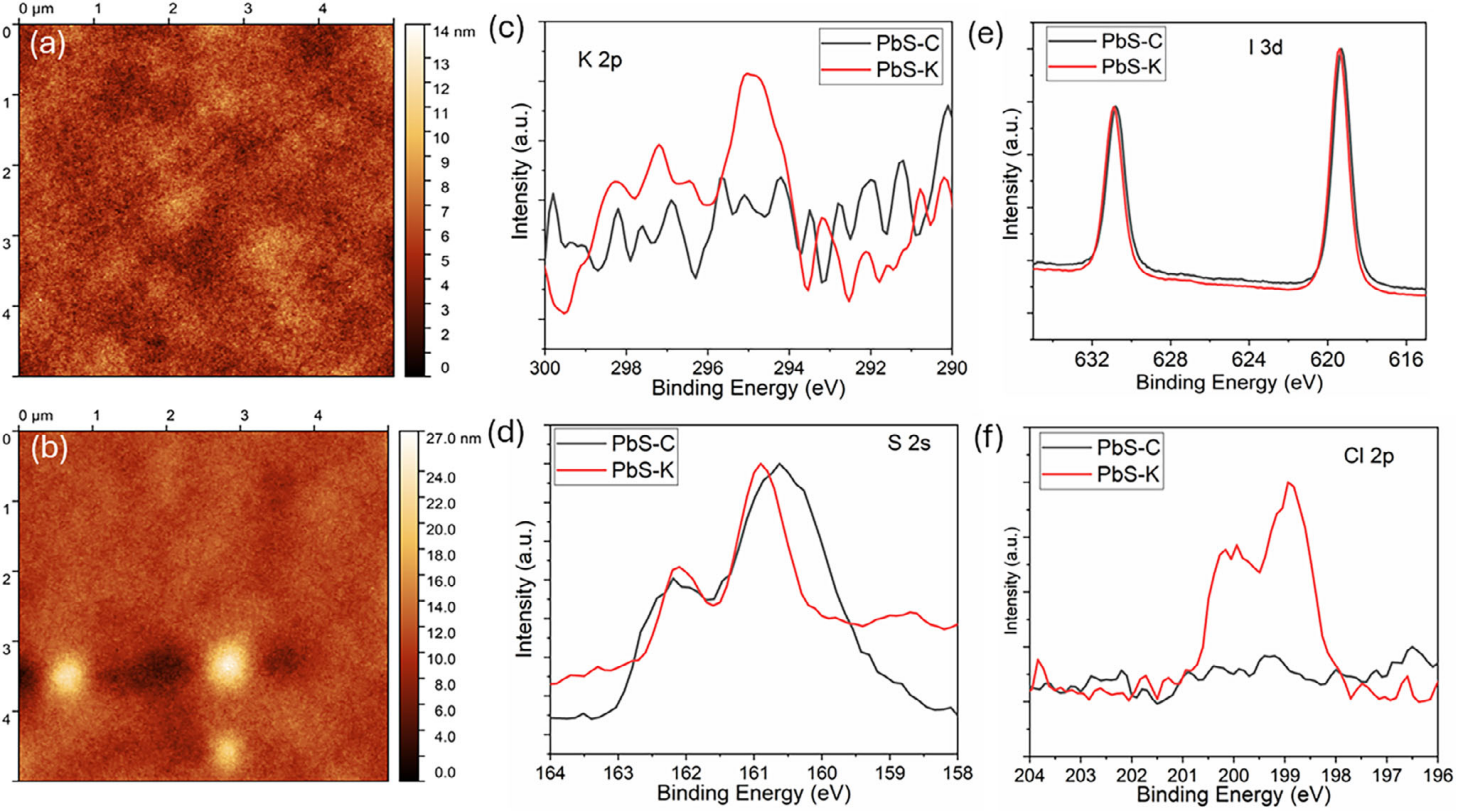
AFM images of (a) PbS‐K and (b) PbS‐C films. Core XPS spectra of (c) Pb 4f, (d) S 2s, (e) K 2p, and (f) Cl 2p for PbS‐K and PbS‐C samples.

The TEM and TRPL results indicate that PbS‐K CQDs outperform the other two samples in terms of photophysical properties and colloidal stability. Therefore, subsequent discussion focuses on the comparative analysis between PbS‐K and PbS‐C. Firstly, atomic force microscopy (AFM) was employed to examine the surface morphology of the CQD films, providing insight into their dispersion uniformity. As shown in Figure 4a,b, the PbS‐K film exhibits a much smoother surface with an average roughness of 1.6 nm, compared to 7.6 nm for the PbS‐C film. This substantial improvement should be attributed to the enhanced colloidal dispersion enabled by the KCl additive [47, 48]. XPS analysis further confirmed the presence of KCl residues in the PbS‐K sample, with clear K 2p and Cl 3d core‐level signals, which were absent in PbS‐C (Figure 4c,d). More importantly, the Pb 4f peaks of PbS‐K exhibited a slight shift toward higher binding energy, suggesting stronger Pb─Cl coordination. Simultaneously, the S 2s peak in PbS‐K was narrower and more symmetric, indicating that surface oxidation of sulfur was effectively suppressed (Figure 4e,f). In short, these results demonstrate that KCl incorporation not only improves film smoothness and colloidal uniformity but also passivates surface defects and inhibits oxidation, thus markedly enhancing the chemical and structural stability of the CQD films [49, 50].

**FIGURE 5:**
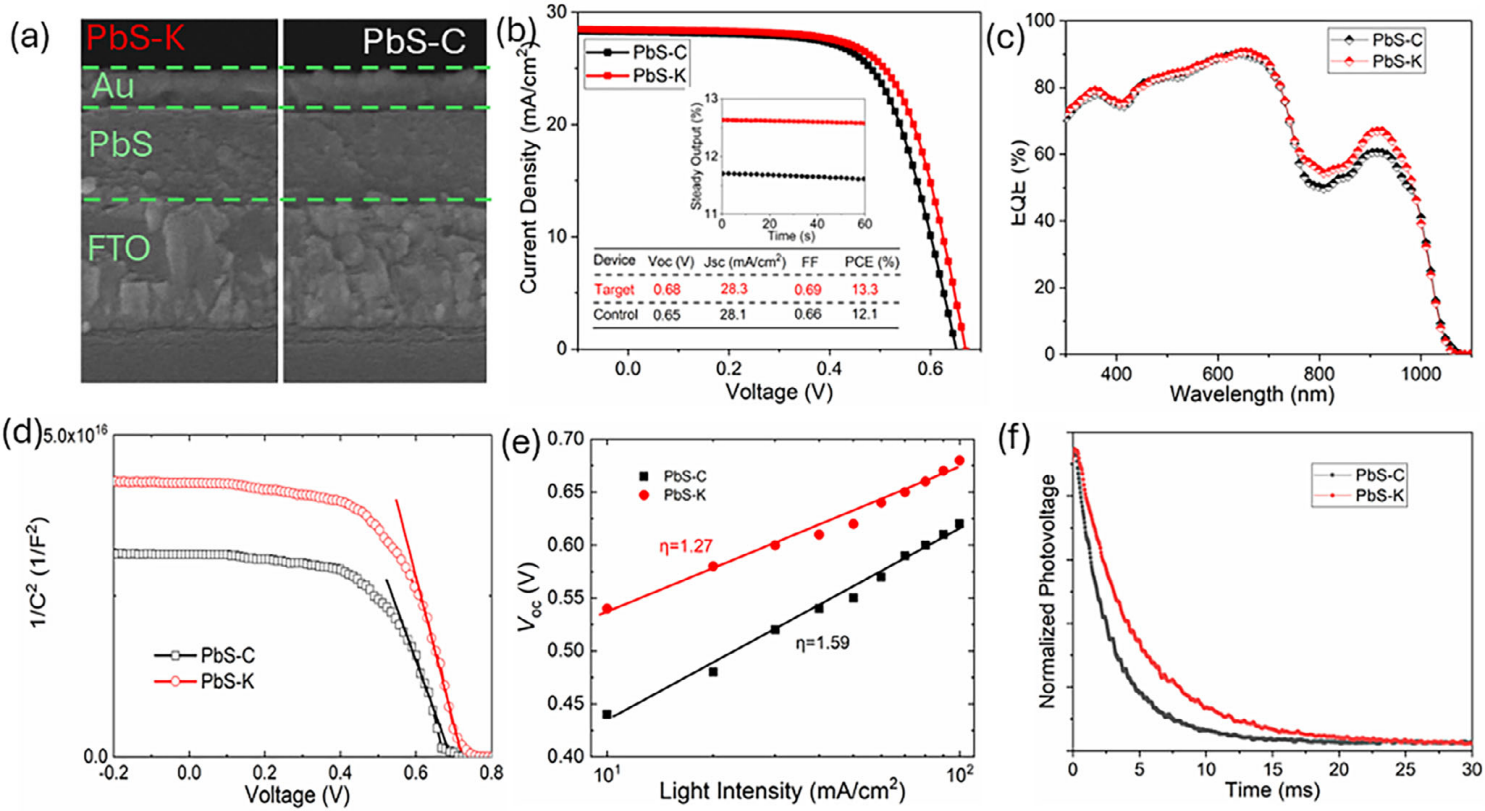
Device characterizations of both types of PbS CQD solar cells. (a) Cross‐sectional SEM images of both types of complete devices. (b) Current density–voltage (*J*–*V*) characteristics. (c) EQE spectra. (d) Capacitance–voltage profiles. (e) Open‐circuit voltage (*V*_oc_) as a function of light intensity. (f) Transient photovoltage (TPV) decay curves of PbS‐K and PbS‐C devices.

Two batches of solar cells were fabricated using PbS‐K and PbS‐C CQDs dispersed in DMF, following a device architecture of FTO/SnO_2_/PbS‐PbI_2_/PbS‐EDT/Au. In this configuration, the chemical‐bath‐deposited SnO_2_ layer serves as the electron transport layer, PbS CQDs function as the active layer, hot‐injection PbS CQDs treated with ethanethiol (EDT) act as the hole transport layer, and thermally evaporated Au serves as the anode. As shown in Figure 5a, cross‐sectional SEM images confirm that both devices exhibit uniform layer thicknesses and consistent morphology. The PbS‐K devices achieved a champion PCE of 13.3%, outperforming the 12.1% obtained from PbS‐C devices with an active area of 0.0314 cm^2^, as determined by current density–voltage (*J–V*) measurements under 1‐sun illumination (Figure 5b). Moreover, the PbS‐K cells maintained a steady output PCE of 12.6% over 60 s at the maximum power point, while the PbS‐C cells started at 11.5% and exhibited a slight decline, as shown in the inset of Figure 5b. For comparison, solar cells fabricated with the PbI_2_‐W precursor only achieved a PCE of 11% (see Figure S3a), which is significantly lower than those of both PbS‐K and PbS‐C devices, thus ruling out discussion of this type of device in the following sections.

Both PbS‐K and PbS‐C devices were tested under forward and reverse voltage scans to evaluate hysteresis behavior (Figure S3b), and both exhibited negligible hysteresis, confirming reliable charge transport and minimal interfacial trapping. The two devices displayed comparable external quantum efficiency (EQE) responses in the 300–780 nm region; however, the PbS‐K device exhibited a notably higher EQE in the extended range of 780–1000 nm (Figure 5c), consistent with enhanced near‐infrared photon harvesting. Statistical analysis across 24 devices of each type (Figure S3c) revealed average PCEs of 12.6% for PbS‐K and 11.4% for PbS‐C, highlighting the superior reproducibility and efficiency enabled by KCl‐assisted PbI_2_. Notably, the PbS‐K device performance approaches the record efficiency (certified 13.4%) achieved by directly synthesized PbS CQD solar cells, which require additional post‐surface treatments with commercial PbI_2_ [42]. This enhancement is attributed to the flatter energy bands and enhanced film uniformity in PbS‐K devices, which collectively suppress carrier trapping and nonradiative recombination [51].

Capacitance–voltage (*C*–*V*) measurements revealed built‐in potentials of 0.73 V for the PbS‐K device and 0.67 V for the PbS‐C (Figure 5d), consistent with the higher open‐circuit voltage observed in *J*–*V* measurements. To further probe recombination mechanisms, the dependence of *V*_oc_ on light intensity was analyzed using the relation *V*oc ∝ *η kT*/*q*, where *k* is the Boltzmann constant, *T* is the absolute temperature, and *q* is the elementary charge. A larger *η* value indicates stronger defect‐mediated recombination. The calculated *η* values were 1.27 for the PbS‐K devices and 1.59 for the PbS‐C one, suggesting fewer trap states in the former. This trend was further corroborated by transient photovoltage (TPV) decay measurements: the PbS‐K device exhibited a carrier lifetime of 46 µs, more than twice that of the PbS‐C device (21 µs). The prolonged lifetime reflects a slower carrier recombination rate and aligns well with the enhanced PCE and superior interfacial quality of the PbS‐K solar cells.

The long‐term stability of the devices was further evaluated under ambient conditions (25°C and 70% RH) without encapsulation and light soaking, and their photovoltaic performances were monitored over time. As shown in Figure S3d, the normalized PCE of the PbS‐K device initially increased to 1.15 after 3 d of storage, likely due to interfacial defect healing, and then gradually stabilized at 0.98 after 60 days. In contrast, the PbS‐C device showed only a slight rise to 1.08 over 3 days, but then degraded markedly to 0.82 after 60 days. This pronounced stability enhancement demonstrates the beneficial role of residual KCl in mitigating oxidation and suppressing defect evolution, in good agreement with previously reported K‐induced stabilization effects in PbS CQD systems [34, 35].

To further optimize the ratios of KCl to PbI_2_ during the recrystallization, three types of PbS QD solar cells fabricated using the recycled PbI_2_ with KCl:PbI_2_ ratios of 0.5:100, 1:100 and 2:100. Table S2 displays the statistics of three types of solar cells, which shows the average efficiencies are 11.6%, 12.6%, and 10.5% respectively, indicating that the optimal ratio is 1:100.

Moreover, the same KCl ratio was added to the PbI_2_‐C DMF solution, which was then recrystallized with IPA for material characterization and device fabrication. As shown in Figure S4a, the SEM image displays that the grain size of recrystallized PbI_2_‐C with KCl is similar to that of recycled PbI_2_–K (1:100). Elemental mapping measurements indicated the atomic weights of I, Pb, and K with 66.13%, 32.95%, and 0.92%, respectively, which are consistent with the stoichiometric ratios (see Figure S4b–d). Subsequently, the recrystallized PbI_2_‐C with KCl treatments was used to synthesize PbS QDs, which were then employed to fabricate solar cells under the same conditions, including device structure and fabrication procedures. As shown in Figure S4e,f, solar cells of PbS‐C with KCl achieved champion and average efficiencies of 13.0% and 12.4%, respectively, which are both lower than those fabricated with recycled PbS‐K (13.3% and 12.6%).

The production cost of PbS CQD solar cells has been significantly reduced by developing scalable blade‐coating techniques that enable large‐area fabrication using directly synthesized PbS CQDs from commercial PbI_2_ sources [42]. Building upon this progress, our work achieves a further breakthrough in cost reduction by utilizing recycled PbI_2_ extracted from spent lead‐acid batteries. This sustainable approach not only minimizes material and energy consumption but also yields colloidally stable, high‐quality PbS CQDs suitable for scalable device manufacturing. Beyond the PbS system, this additive‐assisted recycling strategy can be further generalized to other Pb‐based semiconductors. To verify its universality, CsPbI_3_ quantum dots were synthesized using the same KCl‐assisted PbI_2_ precursor and systematically compared with those derived from commercial sources.

### 2.3 Lead‐Based Halide Perovskite Quantum Dots Prepared from Recycled PbI_2_

Halide perovskite QDs have emerged as a revolutionary class of materials for next‐generation optoelectronics, functioning as efficient light absorbers and emitters owing to their near‐unity photoluminescence quantum yield (PLQY) and highly tunable emission spanning the visible to near‐infrared range through precise control of composition and size [52, 53, 54, 55, 56, 57, 58, 59, 60]. To extend the additive‐assisted recycling strategy beyond PbS CQDs, PbI_2_‐K and PbI_2_‐C were employed to synthesize CsPbI_3_‐K and CsPbI_3_‐C QDs via a hot‐injection route following our previously reported protocol (see Experimental Section for details).

**FIGURE 6:**
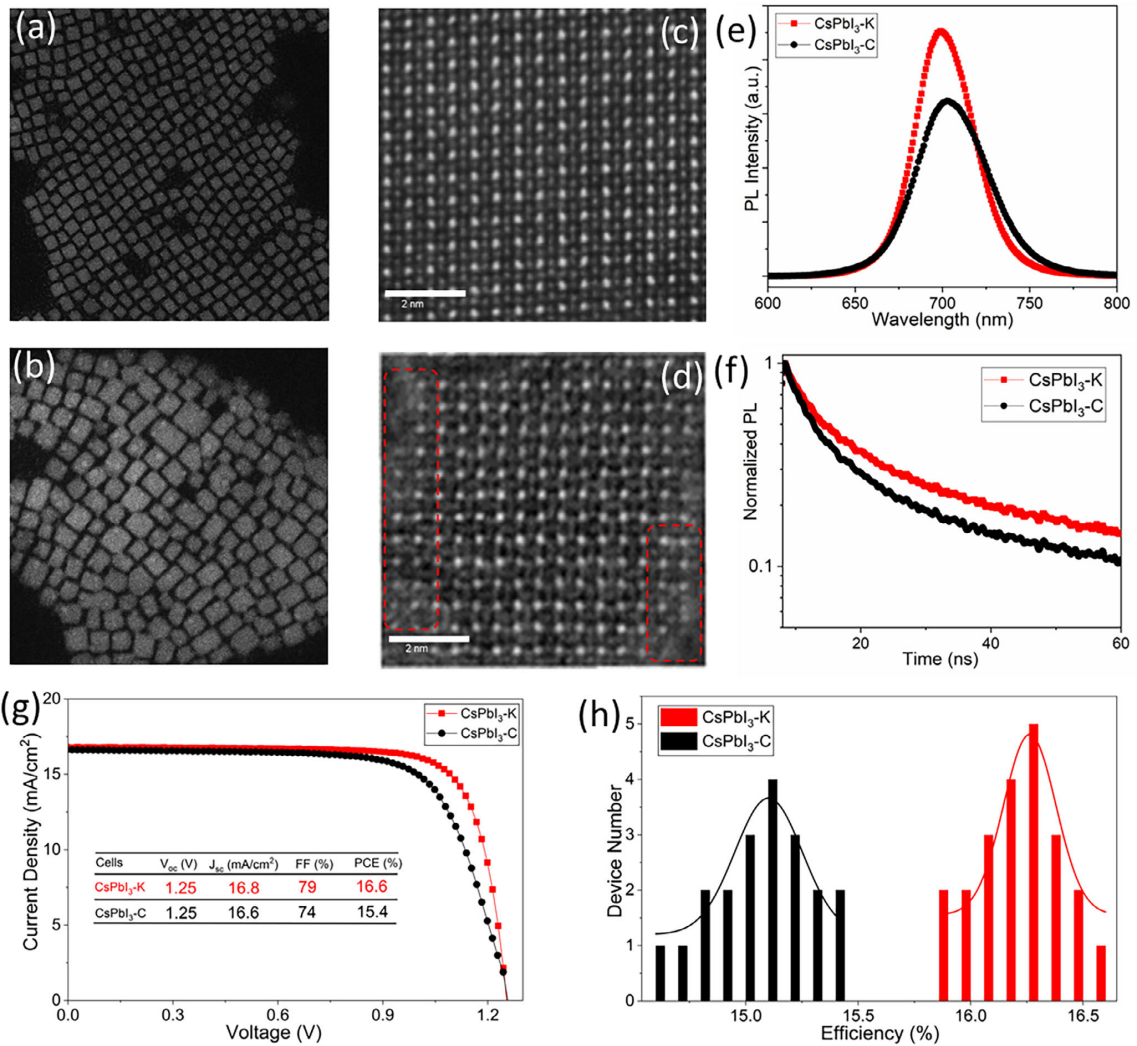
Aberration‐corrected scanning TEM images of (a) CsPbI_3_‐K QDs and (b) CsPbI_3_‐C QDs. High‐resolution atomic structures of (c) CsPbI_3_ QDs‐K and (d) CsPbI_3_‐C QDs, with red circles highlighting edge regions exhibiting enhanced crystallinity. (e) Steady‐state PL and (f) TRPL decay of both CsPbI_3_ QDs. (g) Current density–voltage curves and (h) device efficiency distribution of solar cells of CsPbI_3_ QDs‐K and CsPbI_3_‐C QDs.

Aberration‐corrected scanning TEM (Figure 6a,b) revealed that CsPbI_3_‐K QDs exhibit a significantly narrower size distribution compared with their control counterparts. At the atomic level, microstructural analysis further confirmed that CsPbI_3_‐K QDs possess more intact lattice fringes, particularly at edge regions (highlighted by red circles in Figure 6c,d), implying that the KCl additive improves crystallization and suppresses surface degradation [61]. Moreover, steady‐state and time‐resolved PL measurements show that CsPbI_3_‐K QDs deliver markedly higher PL intensity and longer carrier lifetimes than CsPbI_3_‐C QDs, indicating reduced defect density and nonradiative recombination, as shown in Figure 6e,f [62]. In addition, two batches of solar cells with the device structure FTO/SnO_2_‐CBD/CsPbI_3_/Spiro/Au were fabricated, using CsPbI_3_‐K and CsPbI_3_‐C CQDs as the active layers for efficiency comparison. The champion efficiencies are 16.6% and 15.4%, and the average efficiencies are 16.3% and 15.2% for CsPbI_3_‐K and CsPbI_3_‐C solar cells, respectively (see Figure 6g,h). Device performance comparison further confirmed the benefit of KCl introduction. These findings are consistent with those observed for PbS CQDs, reinforcing the conclusion that the incorporation of KCl into recycled PbI_2_ provides a general benefit for improving the structural and optoelectronic properties of lead‐based QDs.

## 3 Conclusion

In summary, high‐purity PbI_2_ was successfully recovered from spent lead‐acid batteries via an energy‐efficient KCl‐assisted recrystallization route, achieving a production cost of only ∼1 USD g^−1^, approximately 10% of the price of commercial sources. The introduction of KCl effectively promoted PbI_2_ crystallization, facilitated impurity removal, and left beneficial residual species that improved the chemical and structural quality of the recycled precursor. The resulting PbS CQDs exhibited markedly enhanced physicochemical characteristics, including more uniform particle size, superior colloidal stability, and prolonged carrier lifetime. Leveraging these advantages, PbS CQD solar cells delivered a champion PCE of 13.3%, outperforming devices fabricated from commercial (12.1%) and additive‐free recycled PbI_2_ (11.0%). Likewise, CsPbI_3_ CQDs derived from KCl‐assisted PbI2 demonstrated improved monodispersity, surface integrity, and photophysical stability, and the solar cell delivered a champion efficiency of 16.6% that outperformed the 15.4% efficiency in the counterpart cells, confirming the generality of this approach across multiple Pb‐based systems. This work pioneers an additive‐assisted metallurgical recycling strategy that integrates green chemistry and semiconductor material design, providing a scalable, low‐cost, and sustainable pathway to convert industrial Pb waste into device‐grade electronic materials. The demonstrated methodology not only reduces the environmental footprint of Pb sourcing but also sets a paradigm for future sustainable manufacturing of high‐performance quantum dot and perovskite optoelectronics.

## Author Contributions

J.L. conducted the experiments and wrote the original manuscript, and Q.Z. helped with project design and manuscript writing. L.L. and S.Y. performed material measurements. S.D., G.S., and K.L. conducted TEM tests. S.H. offered support for solar cell fabrication. X.G. acknowledges the support from the Macquarie University Research Fellowship (MQRF) and Australian Research Council (DE260100466). L.H. acknowledges the Australian Research Council (DE230101711). X.G., F.L., D.C., and L.H. led the project. All authors discussed the results and commented on the manuscript.

## Funding

Australian Research Council DE260100466, DE230101711

## Conflicts of Interest

The authors declare no conflicts of interest.

## Supporting information




**Supporting File**: advs74565‐sup‐0001‐SuppMat.docx

## Data Availability

The data that support the findings of this study are available from the corresponding author upon reasonable request.
