# Supplementary material for: Sustainable KCl‐Assisted PbI2 Recycling for High‐Performance Quantum Dot Solar Cells
Source: Adv Sci (Weinh). 2026 Feb 21;13(19):e22107. doi: 10.1002/advs.202522107 (PMC13045450; doi:10.1002/advs.202522107)
Supplement: Supplementary file 1 — Supporting File: advs74565‐sup‐0001‐SuppMat.docx [file ADVS-13-e22107-s001.docx]

Supporting Information

**Sustainable KCl-Assisted PbI_2_ Recycling for High-Performance Quantum Dot Solar Cells**

*Jihong Lan, Qiang Zeng, Xinwei Guan^*^, Linhong Li, Songyan Yin, Sandhuli S Hettiarachchi Dehigaspitiya, Guozheng Shi, Kou Li, Shujuan Huang, Fangyang Liu^*^, Dewei Chu^*^, Long Hu^*^*

**Experimental Section**

**1. Chemicals**

Isopropanol (IPA, 99.7%), H_2_O_2_ (30%), KCl (99.9%), Na_2_CO_3_ (99.9%), toluene (99.5%), and acetic acid (98.0%) were purchased from General-Reagent, Sinochem. Dimethylformamide (DMF, 99.8%), lead iodide (PbI_2_, 99.999%), cesium carbonate (Cs_2_CO_3_, 99%), octadecene (ODE, 90%), oleic acid (OA, 90%), oleylamine (OLA, 70%), hexane (anhydrous, 95%), hexane (anhydrous, ≥99%), methyl acetate (MeOAc, anhydrous, 99.5%), *N*,*N*′-diphenylthiourea (DPhTA, 98%), butylamine (BTA, 99.5), urea, hydrochloric acid (HCl, 37 wt. % in water), thioglycolic acid (TGA, 98%), SnCl_2_·2H_2_O (>99.995%), PbO (99.99%), and hexamethyldisilathiane (TMS) were purchased from Sigma-Aldrich without further purification unless mentioned. Glass/ITO and PET/ITO were purchased from the Zhuhai Kaivo Optoelectronic Technology Co., Ltd.

**2. Materials synthesis**

**2.1 PbS CQD synthesis**

2.3 g of PbI_2_ with the additive and 0.238 g of DPhTA are fully dissolved in 9 ml of DMF under stirring at room temperature in a glove box filled with N_2_. The precursor solution in a two-neck flask was transferred to ambient conditions under N_2_ protection and cooled in an ice bath (0 °C). 1 mL of BTA was then quickly injected into the above precursor solution for 10 min reaction to convert PbI_2_ into PbS CQDs. After the reaction, 25 mL of toluene was added to the crude CQD solution, and the mixture was centrifuged at 8500 rpm for 3 min to remove aggregates and oversized nanocrystals. Finally, 10 mL more toluene was added to the supernatant to extract the CQDs by centrifuging under the same conditions, and the CQDs were redispersed in DMF for characterizations and device fabrication. Both types of PbS CQDs were synthesized under identical conditions, differing only in the presence or absence of the KCl additive.

**2.3 CsPbI_3_ CQDs synthesis**

Two three-neck flasks were prepared to synthesize two batches of CsPbI_3_ quantum dots using PbI_2_ with and without the additive. For each batch of CsPbI_3_ QDs, all conditions were identical except for the additive. 0.5 g of PbI_2_, 2.5 mL of OA, and 25 mL of ODE were loaded into a 100 mL three-neck flask and vacuum-pumped under stirring at 100 °C for 1 h. Then, 2.5 ml of oleylamine (OLA) was injected into the flask. After PbI_2_ was completely dissolved, the temperature was increased to 160 °C under a N_2_ flow. 4 mL of the Cs-oleate OA (0.0625 M) precursor was swiftly injected into the reaction mixture, and the solution was quenched in an ice bath. The crude CsPbI_3_ CQD solution was evenly divided into 3 centrifugation tubes, and methyl acetate was added to the tubes at a volume ratio of 1:2 (QD solution: methyl acetate). Subsequently, QD precipitate was extracted by centrifugation at a speed of 8000 rpm for 3 min. All QD precipitate in 3 tubes was dispersed with 3 ml hexane, then precipitated by adding 4.5 ml methyl acetate, and centrifuged again at 8000 rpm. Finally, CQDs were redispersed into octane for characterizations.

**2.3 CsPbI_3_ CQDs synthesis**

Two three-neck flasks were prepared to synthesize two batches of CsPbI_3_ quantum dots using PbI_2_ with and without the additive. For each batch of CsPbI_3_ QDs, all conditions were identical except for the additive. 0.5 g of PbI_2_, 2.5 mL of OA, and 25 mL of ODE were loaded into a 100 mL three-neck flask and vacuum-pumped under stirring at 100 °C for 1 h. Then, 2.5 ml of oleylamine (OLA) was injected into the flask. After PbI_2_ was completely dissolved, the temperature was increased to 160 °C under a N_2_ flow. 4 mL of the Cs-oleate OA (0.0625 M) precursor was swiftly injected into the reaction mixture, and the solution was quenched in an ice bath. The crude CsPbI_3_ CQD solution was evenly divided into 3 centrifugation tubes, and methyl acetate was added to the tubes at a volume ratio of 1:2 (QD solution: methyl acetate). Subsequently, QD precipitate was extracted by centrifugation at a speed of 8000 rpm for 3 min. All QD precipitate in 3 tubes was dispersed with 3 ml hexane, then precipitated by adding 4.5 ml methyl acetate, and centrifuged again at 8000 rpm. Finally, CQDs were redispersed into octane for characterizations.

**3. Device fabrication**

**3.1 CBD-SnO_2_ deposition**

A chemical bath deposition (CBD)-SnO2 layer serving as an ETL was deposited on patterned FTO substrates with a 2-min plasma treatment. 625 mg of urea and 137.5 mg of SnCl_2_·2H_2_O were dissolved in 50 mL of deionized water, followed by the addition of 62 μL of HCl and 12.5 μL of TGA to form a uniform solution under stirring. The prepared solution was loaded onto a glass reaction vessel. The treated FTO substrate was placed vertically in the glassware, and the reaction was maintained at 90 °C for 4.5 h. After the reaction was complete, the FTO/SnO2 substrates were cleaned by sonication in deionized water and IPA for 5 min each, and then annealed in air at 170 °C for 60 min.

**3.2 Device construction**

PbS CQD solution (700 mg mL^-1^) was spin-coated on FTO/SnO_2_ substrates at 2000 rpm for 40 s, followed by annealing at 70 °C for 10 min in a N_2_-filled glovebox. PbS CQD films formed from PbS CQD ink were transferred into ambient conditions. OA-capped PbS CQDs solution in octane was spin-coated on annealed PbS CQDs films after cooling to room temperature. Then, 0.2% EDT acetonitrile solution was dropped for ligand exchange, followed by pure acetonitrile for rinsing. This procedure was repeated 2 times to produce HTLs. Finally, 100 nm gold was thermally deposited to form the electrode. The CsPbI_3_-K and CsPbI_3_-C CQDs dispersed into octane solvent with a concentration of 70 mg/ml were spin-coated on SnO_2_-CBD/FTO substrates; then treated with methyl acetate in a dry box. This procedure was repeated 4 times to yield a film with a thickness of 350 nm; Spiro solution was spin-coated to produce a hole transport layer; finally, 100 nm gold was thermally evaporated to construct a complete device.

**4. Characterization**

The PL spectra measurements were performed at room temperature using a custom laser PL spectroscopy system (Crystal Laser, Model BLC-050-405). The laser pulse width was 130 fs, and the repetition rate was 100 MHz. The excitation wavelength for both PL and TRPL measurements is 600 nm. TEM measurements were performed by a JEOL JEM-2010 and a JEOL JEM-F200 operated at 200 kV. XPS measurements were conducted by a VG ESCALAB MK2 system with monochromatized Al Kα radiation under a pressure of 5.0 × 10^−7^Pa. XRD (Panalytical Empyrean I system, Cu Kα radiation with a wavelength of 0.154 nm) and SEM (FEI Nova Nano 450) measurements were used to measure the structures of PbI_2_ powder.

PbS CQD solar cell devices were tested on a Newport AAA solar simulator (94023A-U) with a Xenon lamp at room temperature using a Keithley 2400 (I-V) digital source meter. The intensity of the solar simulator was calibrated to 100 mW/cm2 AM 1.5 G using a standard silicon cell with a KG-5 filter. J-V scans were measured from forward bias to reverse bias step and from reverse bias to forward (−0.8 V → 0.8 V, step 0.0125 V, scan rate: 0.1 V/s). For the light intensity dependence measurement, neutral density filters with optical densities ranging from 0.1 to 1, in 0.2 increments, were used.

**5. Estimated recycling cost of PbI_2_**

Briefly, a spent 20 Ah lead-acid battery (purchased for recycling at approximately USD 4.4) was disassembled to yield ~2.5 kg of used lead paste, corresponding to a raw material cost of USD ~0.0018 per gram. In our experiment, 10 g of waste lead paste was processed to yield 8 g of PbI_2_. For the lab-scale experiment, we estimate a total operator time of approximately 2 hours (including battery disassembly, material handling, and the synthesis process). Assuming an average hourly operator cost of USD 3.0 (a standard rate for a pilot-scale facility), the total operator cost for this batch would be USD 6.0. Amortizing this over the 8 grams of synthesized PbI₂ adds USD 0.72 per gram. Including additional reagent costs of approximately USD 7.2, the total production cost of the recycled PbI_2_ is estimated to be USD ~1.65 per gram which is substantially lower than that of currently available products on the market (~USD 10.0 per gram from Sigma-Aldrich).

**Table S1.** ICP-MS spectra of three types of PbI_2_ (weight%; ----: not detected).

| **Element** | **PbI_2_-W** | **PbI_2_-K** | **PbI_2_-C** | **Element** | **PbI_2_-W** | **PbI_2_-K** | **PbI_2_-C** |
| --- | --- | --- | --- | --- | --- | --- | --- |
| Ag | 0.0005 | 0.0002 | <0.0001 | Mn | 0.0015 | 0.0005 | <0.0001 |
| Al | 0.0010 | 0.002 | 0.0003 | Mo | ---- | ---- | ---- |
| As | 0.0001 | 0.0001 | 0.0001 | Na | 0.0044 | 0.0048 | 0.0068 |
| B | 0.0006 | 0.0001 | 0.0002 | Ni | <0.0001 | <0.0001 | <0.0001 |
| Ba | ---- | ---- | ---- | P | <0.0001 | <0.0001 | 0.0001 |
| Be | ---- | ---- | ---- | Cl | 0.0005 | 0.076 | 0.0004 |
| Ca | 0.0015 | 0.0007 | 0.0028 | S | 0.0010 | 0.0009 | 0.0008 |
| Cd | <0.0001 | <0.0001 | <0.0001 | Sb | 0.0034 | 0.0004 | 0.0006 |
| Ce | ---- | ---- | ---- | Sc | ---- | ---- | ---- |
| Co | ---- | ---- | ---- | Se | <0.0001 | ---- | <0.0001 |
| Cr | <0.0001 | <0.0001 | ---- | Si | 0.0051 | 0.0011 | 0.0021 |
| Cu | 0.0002 | 0.0001 | <0.0001 | Sn | <0.0001 | <0.0001 | <0.0001 |
| Fe | 0.0015 | 0.0007 | 0.0006 | Sr | <0.0001 | ---- | <0.0001 |
| Hg | <0.0001 | ---- | <0.0001 | Ti | <0.0001 | ---- | <0.0001 |
| K | 0.0006 | 0.042 | 0.0004 | V | <0.0001 | ---- | ---- |
| La | ---- | ---- | ---- | Y | ---- | ---- | ---- |
| Li | ---- | ---- | ---- | Zn | 0.0009 | 0.0004 | 0.0005 |
| Mg | 0.0003 | 0.0002 | 0.0006 | Zr | ---- | ---- | ---- |

**Table S2.** Device statistics for three types of solar cells fabricated with PbI_2_ and varying KCl ratios.

| **The ratios** | **V_oc_ (V)** | **J_sc_ (mA/cm^2^)** | **FF (%)** | **PCE (%)** |
| --- | --- | --- | --- | --- |
| (0.5:100) | 0.64±0.3 | 27.8±1.2 | 65±3 | 11.6±0.4 |
| (1:100) | 0.67±0.3 | 28.1±1.0 | 67±2 | 12.6±0.4 |
| (2:100) | 0.62±0.4 | 27.4±1.4 | 62±4 | 10.5±0.5 |


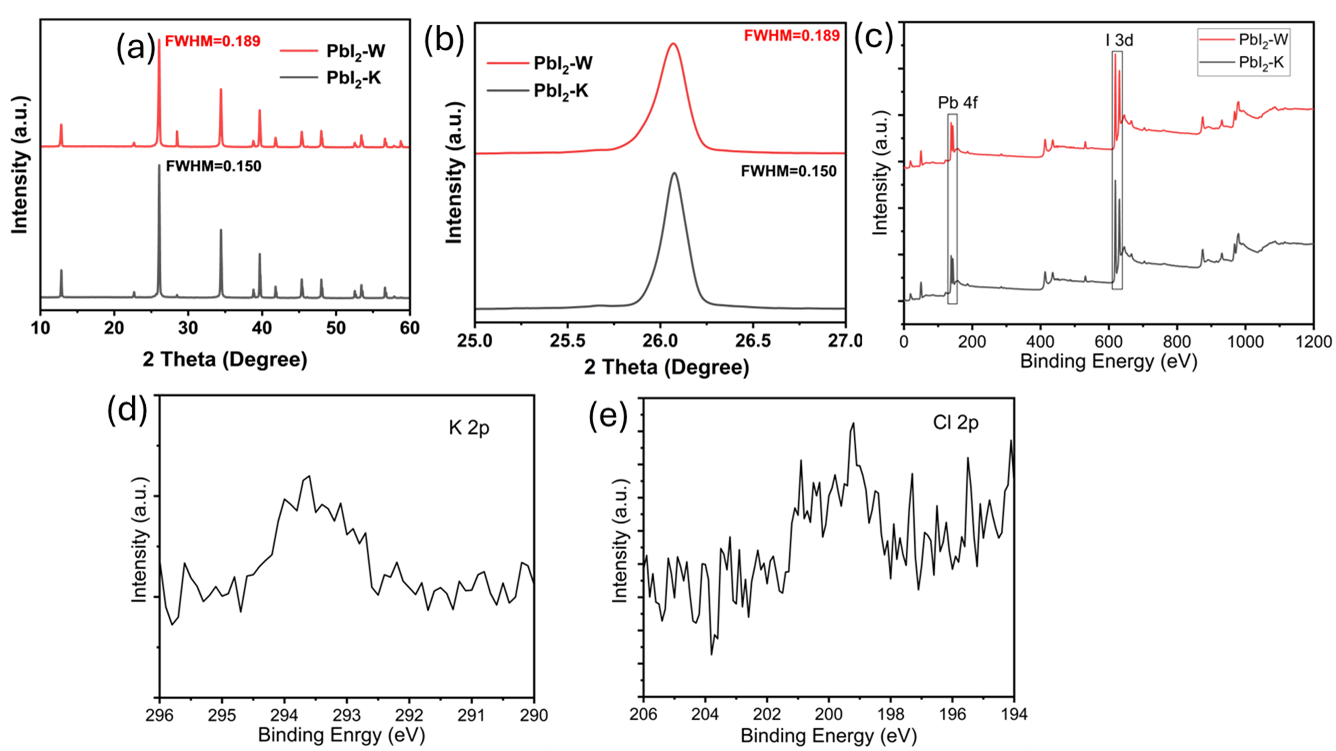


**Figure S1.** (a) XRD patterns (b) enlarged XRD pattern of (202) diffraction peak of PbI_2_ with and without KCl introduction. (c) XPS whole spectra of PbI_2_ with and without KCl introduction. Core XPS spectra of (d) K 2p and (e) Cl 2P.


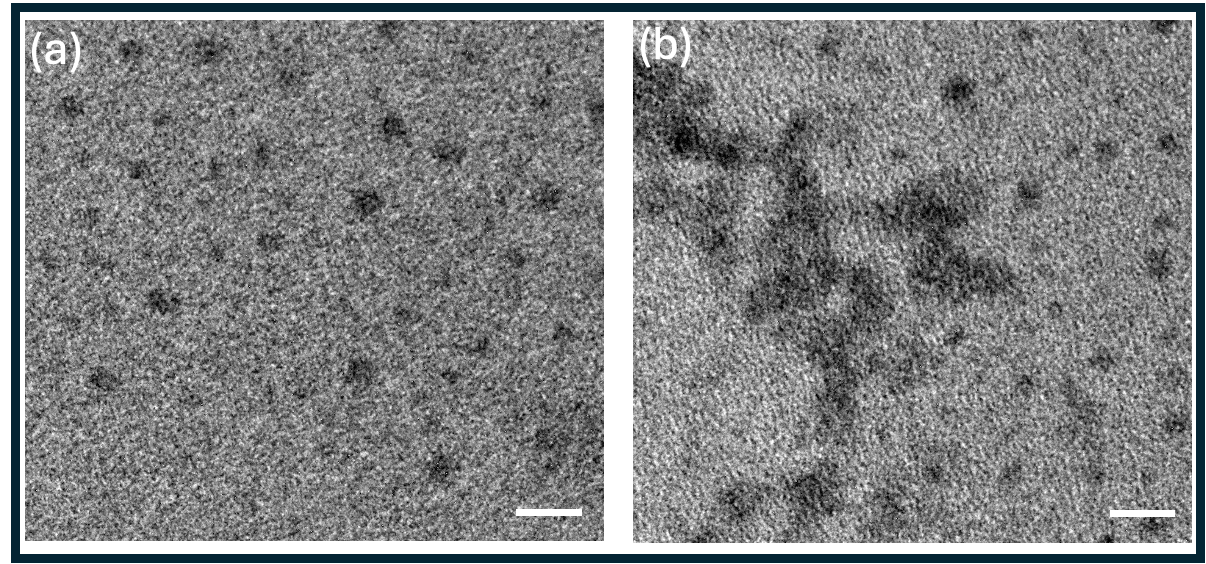


**Figure S2.** TEM images of (a) fresh PbS-C QDs and (b) PbS-C QDs with post-synthetic treatment after 40-hour storage (Scale bar: 5 nm).


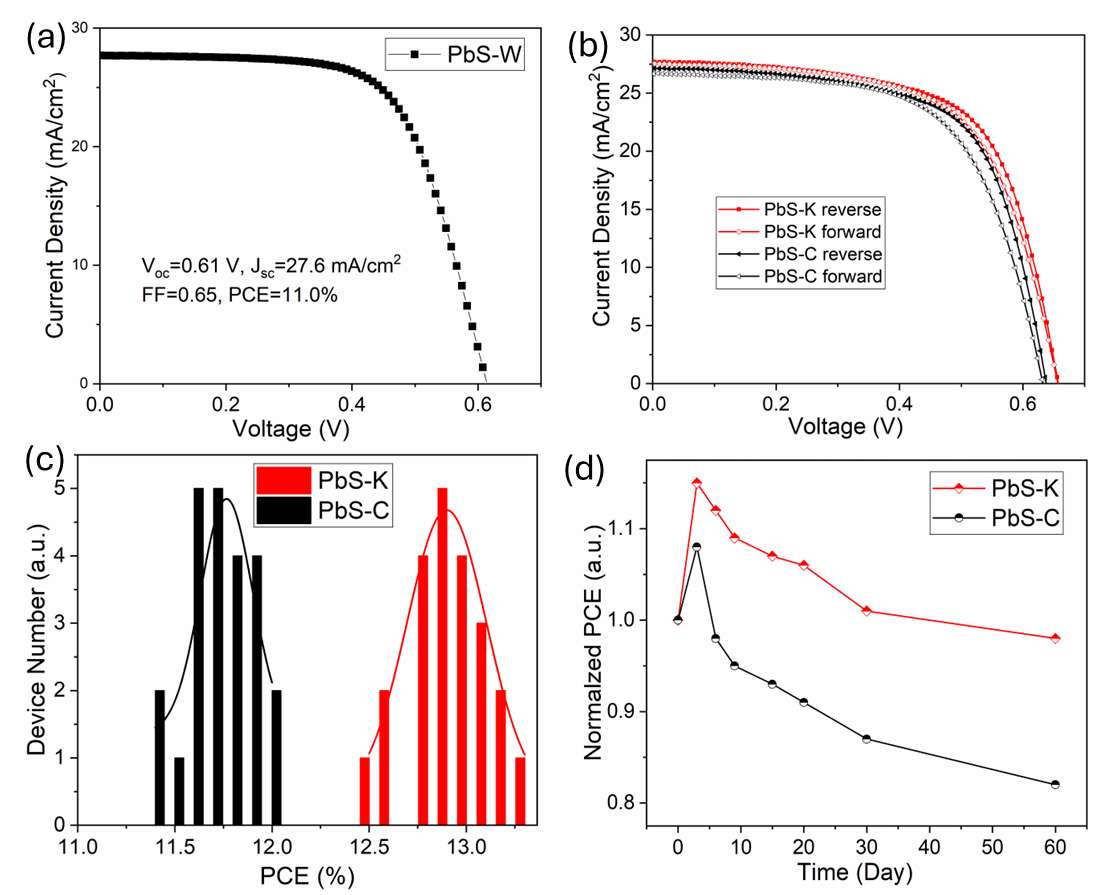


**Figure S3.** (a) J-V curve of PbS-W solar cells. (b) J-V curves of target and control devices under reverse and forward scans. (c) Device efficiency statistics (d) Device stability of PbS-K and PbS-C solar cells.


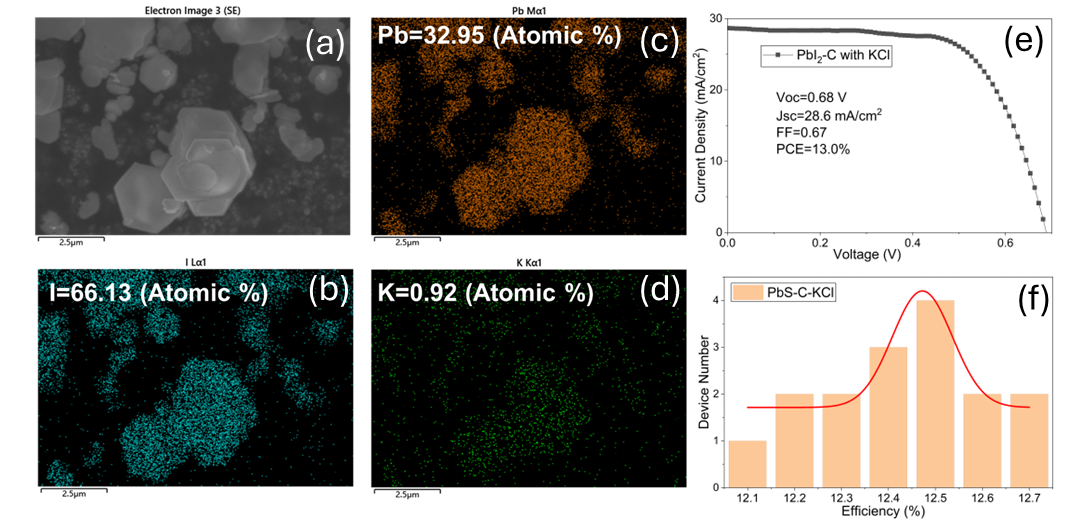


**Figure S4.** (a) SEM image and EDS elemental mapping of (b) I, (c) Pb, and (d) K for commercially purchased PbI_2_ after KCl-assisted recrystallization (PbI_2_-C + KCl). (e) The champion efficiency and (f) efficiency distribution of PbS QD solar cells fabricated with PbI_2_-C + KCl as the precursor.
